# Supplementary material for: Deletion of wfs1 Impairs Oligodendrocyte Precursor Cells Dorsal Distribution and Myelination Through the wfs1-hmgcs1 Axis in Zebrafish
Source: Biology (Basel). 2026 Aug 16;15(16):1402. doi: 10.3390/biology15161402 (PMC13510151; doi:10.3390/biology15161402)
Supplement: Supplementary file 1 [file biology-15-01402-s001.zip › File S1-Transcriptome sequencing data and differentially expressed genes (DEGs) in wfs1-- zebrafish at 5 dpf/2.KEGG/wfs1KOvsWT/GSEA/wfs1KO/BUTANOATE_METABOLISM/BUTANOATE_METABOLISM(DRE00650).html]

Details for gene set BUTANOATE\_METABOLISM(DRE00650)[GSEA]

|  || Dataset | deg.sample |
| Phenotype | sample.cls |
| Upregulated in class | wfs1KO |
| GeneSet | BUTANOATE\_METABOLISM(DRE00650) |
| Enrichment Score (ES) | 0.5364774 |
| Normalized Enrichment Score (NES) | 1.4562659 |
| Nominal p-value | 0.11133201 |
| FDR q-value | 0.17644744 |
| FWER p-Value | 0.807 |
Table: GSEA Results Summary

  

Fig 1: Enrichment plot: BUTANOATE\_METABOLISM(DRE00650)      
 Profile of the Running ES Score & Positions of GeneSet Members on the Rank Ordered List

  

| PROBE | DESCRIPTION (from dataset) | GENE SYMBOL | GENE\_TITLE | RANK IN GENE LIST | RANK METRIC SCORE | RUNNING ES | CORE ENRICHMENT || 1 | 565235\_1 | aldh5a1 |  |  | 226 | 4.030 | 0.2368 | Yes |
| 2 | 797311 | LOC797311 |  |  | 976 | 2.119 | 0.3416 | Yes |
| 3 | 553401 | hadhaa |  |  | 1608 | 1.593 | 0.4182 | Yes |
| 4 | 394190 | hmgcl |  |  | 2729 | 1.102 | 0.4498 | Yes |
| 5 | 445288 | acads |  |  | 3897 | 0.848 | 0.4645 | Yes |
| 6 | 30643 | acat2 |  |  | 4174 | 0.798 | 0.5041 | Yes |
| 7 | 100000859 | ehhadh |  |  | 4565 | 0.737 | 0.5365 | Yes |
| 8 | 100038790 | zgc:163121 |  |  | 5957 | 0.591 | 0.5286 | No |
| 9 | 571388 | hmgcll1 |  |  | 9312 | 0.346 | 0.4443 | No |
| 10 | 793834 | hadhab |  |  | 10077 | 0.298 | 0.4384 | No |
| 11 | 100329827 | gad1a |  |  | 10096 | 0.296 | 0.4557 | No |
| 12 | 394060 | hmgcs1 |  |  | 11015 | 0.243 | 0.4416 | No |
| 13 | 442923\_1 | oxct1a |  |  | 11274 | 0.229 | 0.4474 | No |
| 14 | 100037356 | bdh1 |  |  | 11416 | 0.221 | 0.4564 | No |
| 15 | 100002035 | acsm3 |  |  | 12623 | 0.158 | 0.4281 | No |
| 16 | 445121 | hadh |  |  | 18086 | -0.080 | 0.2615 | No |
| 17 | 368912 | echs1 |  |  | 18310 | -0.089 | 0.2600 | No |
| 18 | 560273 | oxct1b |  |  | 18736 | -0.110 | 0.2533 | No |
| 19 | 378441 | gad1b |  |  | 19299 | -0.135 | 0.2438 | No |
| 20 | 378968 | abat |  |  | 19919 | -0.162 | 0.2342 | No |
| 21 | 566872 | l2hgdh |  |  | 20836 | -0.206 | 0.2180 | No |
| 22 | 550403 | gad2 |  |  | 21132 | -0.223 | 0.2222 | No |
| 23 | 445290 | acat1 |  |  | 21467 | -0.239 | 0.2262 | No |
| 24 | 393984 | aacs |  |  | 22011 | -0.268 | 0.2254 | No |
| 25 | 442923 | oxct1a |  |  | 24062 | -0.397 | 0.1850 | No |
| 26 | 565235 | aldh5a1 |  |  | 29304 | -1.001 | 0.0812 | No |
Table: GSEA details [plain text format]

  

Fig 2: BUTANOATE\_METABOLISM(DRE00650)      
 Blue-Pink O' Gram in the Space of the Analyzed GeneSet

  

Fig 3: BUTANOATE\_METABOLISM(DRE00650): Random ES distribution      
 Gene set null distribution of ES for **BUTANOATE\_METABOLISM(DRE00650)**
